# A Systematic Review of Complications Following Elective Foot and Ankle Surgery

**DOI:** 10.1002/jfa2.70235

**Published:** 2026-10-18

**Authors:** Lia Spencer, Connor Phillips, Sifat Alam, Kit Ferguson, Richard Feinn, Douglas McHugh, Ewan Kannegieter

**Affiliations:** ^1^ Department of Medical Sciences Frank H. Netter MD School of Medicine Quinnipiac University Hamden Connecticut USA

**Keywords:** elective ankle surgery, elective foot surgery, nonunion, postoperative complications, risk factors, surgical site infection, systematic review

## Abstract

**Introduction:**

Complications following elective foot and ankle surgery are a significant source of morbidity and may impair recovery. Evidence is often procedure specific, limiting a broader understanding. This review aimed to synthesize complication profiles, risk factors, and mitigation strategies across elective procedures.

**Methods:**

PubMed, Scopus, and CINAHL were searched for studies published between 1 January 2020 and 30 June 2025. Eligible studies included adults undergoing elective foot and ankle surgery and reporting postoperative complications. Data were extracted on complication type, incidence, and risk factors, and synthesized narratively due to heterogeneity.

**Results:**

Nine studies (*n* = 20,455) were included. Complication rates varied: infection (0.3%–12.5%), wound healing problems (0.5%–10.0%), nonunion (0.8%–18.7%), hardware removal (3.8%–15.5%), nerve injury (0.2%–10.0%), pain or stiffness (0.8%–18.7%), and systemic complications (3.6%–7.4%). Consistent risk factors included diabetes, obesity, smoking, and peripheral vascular disease. Higher complication rates were associated with revision surgery, prolonged operative time, and multisegment reconstruction.

**Conclusion:**

Complications after elective foot and ankle surgery are common and multifactorial, with consistent risk patterns across procedures. These findings support improved risk stratification, targeted preoperative optimization, and structured postoperative care.

**Abbreviations**
ATR: Achilles tendon rupture
BMI: Body mass index
CRPS: Complex regional pain syndrome
ERAS: Enhanced recovery after surgery
MIS: Minimally invasive surgery
NHLBI: National Heart, Lung, and Blood Institute
ORIF: Open reduction and internal fixation
PRISMA: Preferred Reporting Items for Systematic Reviews and Meta‐Analyses
PROMs: Patient‐reported outcome measures
RCT: Randomized controlled trial
ROM: Range of motion
SSI: Surgical site infection
TAA: Total ankle arthroplasty

## 1 Introduction

Elective foot and ankle surgery encompasses a range of procedures aimed at correcting deformities, alleviating pain, and restoring mobility in patients with diverse musculoskeletal conditions, including hallux valgus, ankle instability, degenerative joint disease, and post‐traumatic sequelae [1]. Advances in surgical techniques, such as minimally invasive approaches (MIS) and improved fixation devices, have expanded patient eligibility and optimized outcomes [2, 3, 4]. Over the past 2 decades, procedural volumes have risen in the United States, the United Kingdom, and other nations, driven by demographic changes, increased participation in sports, and greater recognition of surgical benefits [5, 6]. The anatomical complexity of the foot and ankle, combined with its critical role in weight‐bearing, presents unique postoperative challenges [7, 8]. This complexity not only influences surgical planning and technique but also underlies the diverse spectrum of complications that may occur following these procedures.

Postoperative complications in foot and ankle surgery range from relatively minor issues, such as pain, swelling, superficial wound‐healing delays, scarring, and delayed wound closure, to more severe problems including deep infection, nonunion or delayed union of bone, implant or fixation irritation, deep vein thrombosis (DVT), pulmonary embolism (PE), and complex regional pain syndrome (CRPS). Other recognized complications include recurrence of pathology, transfer pain or secondary pathology through altered gait dynamics, persistent symptoms despite intervention, and the need for return to theater [9, 10, 11]. Infection rates in elective cases range from below 2% in low‐risk patients to over 5% in those undergoing revision surgery or with significant comorbidities, a pattern broadly consistent with broader foot and ankle surgical populations [12, 13, 14]. Certain conditions, such as diabetic foot osteomyelitis, carry additional risk, with treatment failure rates exceeding 20% and major amputation rates between 5% and 7% in some cohorts [15, 16]. Complications often prolong rehabilitation, necessitate reoperation, and impair quality of life [17], while also increasing healthcare costs and resource utilization [18, 19, 20].

Risk factors for postoperative complications are multifactorial. Patient‐related factors include diabetes, obesity, smoking, peripheral vascular disease, and advanced age, all of which have been linked to impaired healing and higher infection risk [21, 22, 23]. Surgical factors such as prolonged operative time, complex reconstructions, and revision procedures also contribute [24, 25, 26]. Microbiological findings can further influence outcomes; for example, isolation of *Pseudomonas* species from bone cultures in diabetic foot cases has been associated with higher failure and amputation rates [27]. Evidence from US and UK studies suggests that optimization of modifiable risk factors preoperatively can reduce the incidence of complications and improve long‐term outcomes [28, 29].

Although many studies have addressed complications in foot and ankle surgery, much of the literature is procedure‐specific, small‐scale, or limited to single‐center series [30, 31]. There is substantial heterogeneity in complication definitions, follow‐up durations, and infrequent outcome reporting, making it challenging to compare results across studies [32]. Systematic reviews to date have tended to focus on individual procedures such as total ankle arthroplasty or bunion correction rather than synthesizing findings across the full range of elective surgeries [33, 34]. A comprehensive synthesis addressing incidence rates, risk factors, and mitigation strategies across all elective foot and ankle procedures remains lacking.

Given the clinical and economic implications of postoperative complications, this review aims to provide an up‐to‐date synthesis of the literature on elective foot and ankle surgery. Specifically, it will (1) describe reported complications and their incidence within the included studies across a range of elective procedures, (2) summarize reported risk factors associated with these complications, and (3) describe reported strategies to reduce risk and improve surgical outcomes, with attention to evidence from both US and UK healthcare contexts.

## 2 Materials and Methods

### 2.1 Search Strategies

A systematic and comprehensive search was conducted using the PubMed, Scopus, and CINAHL databases to identify relevant studies exploring complications following foot and ankle surgery between 1 January 2020 and 30 June 2025. Searches were constructed using four concept groups—(1) procedure, (2) complication, (3) outcome, and (4) study design—combined with AND logic so that results had to satisfy all four concepts simultaneously. Within each concept, synonyms are linked with OR operators and enclosed in parentheses.

The search targeted literature concerning the incidence, associated risk factors, and patient impact of surgery complications. Studies included in this review were required to focus on elective surgery (open and keyhole/minimally invasive) performed on adults (age 18+). The full Boolean expression below was copied and pasted as a single line into the PubMed Advanced Search Builder without quotation breaks between groups, ensuring that parentheses and AND operators were preserved exactly as shown. When executed on 30 June 2025, the search returned 108 records before duplicate removal. Due to ongoing PubMed indexing, the same query now yields 102 records (as of 26 October 2025), which is consistent with normal database updating. Equivalent logic was applied to Scopus and CINAHL using adjusted syntax.

The search strategy in PubMed incorporated the following terms: (“foot surgery” OR “ankle surgery” OR “podiatric surgery” OR “orthopedic foot surgery” OR “elective foot surgery” OR “non‐elective foot surgery” OR “trauma surgery” OR “bunionectomy” OR “ankle arthroplasty” OR “Achilles tendon repair” OR “fracture fixation”) AND (“complications” OR “adverse events” OR “postoperative complications” OR “surgical site infection” OR “hardware failure” OR “nerve injury” OR “vascular compromise” OR “wound dehiscence” OR “thromboembolism” OR “chronic pain syndrome”) AND (“surgical outcomes” OR “treatment failure” OR “reoperation rates” OR “readmission within 30 days” OR “long‐term patient outcomes”) AND (“randomized clinical trial” OR “randomized controlled trial” OR “prospective cohort study” OR “retrospective cohort study” OR “case‐control study”) AND (“English”[Language]).

Duplicates were removed manually by two reviewers (L.S. and C.P.) prior to screening; no automated deduplication software was used. Titles and abstracts were then independently screened by three reviewers (L.S., C.P., and D.M.), each using the ChatGPT‐4o prompt (Appendix 1) as a structured decision aid to apply the pre‐specified inclusion and exclusion criteria [35]. The prompt was not used as an autonomous screening tool; each reviewer applied it independently to support consistent application of the eligibility criteria to every record. Discrepancies between reviewers were resolved by discussion and, where agreement could not be reached, by adjudication from a fourth author (E.K.). For full‐text screening, the same three reviewers independently assessed the eligibility of the 15 retrieved reports against the pre‐specified criteria. The ChatGPT‐4o prompt was applied at this stage as a criteria checklist only; final inclusion decisions were made by the reviewers and not by the AI tool. Discrepancies at the full‐text stage were again resolved by discussion and adjudication (E.K.). The screening prompt (Appendix 1) was designed for title and abstract assessment and was applied at the full‐text stage without adaptation. All AI‐assisted outputs were reviewed and verified by the authors prior to final inclusion decisions.

We structured this review in line with the PRISMA‐ScR reporting framework to maintain transparency and methodological rigor [36]. The study selection pathway is illustrated in a PRISMA flow diagram (Figure 1). The protocol was prospectively registered with PROSPERO (CRD1035192) [37].

**FIGURE 1:**
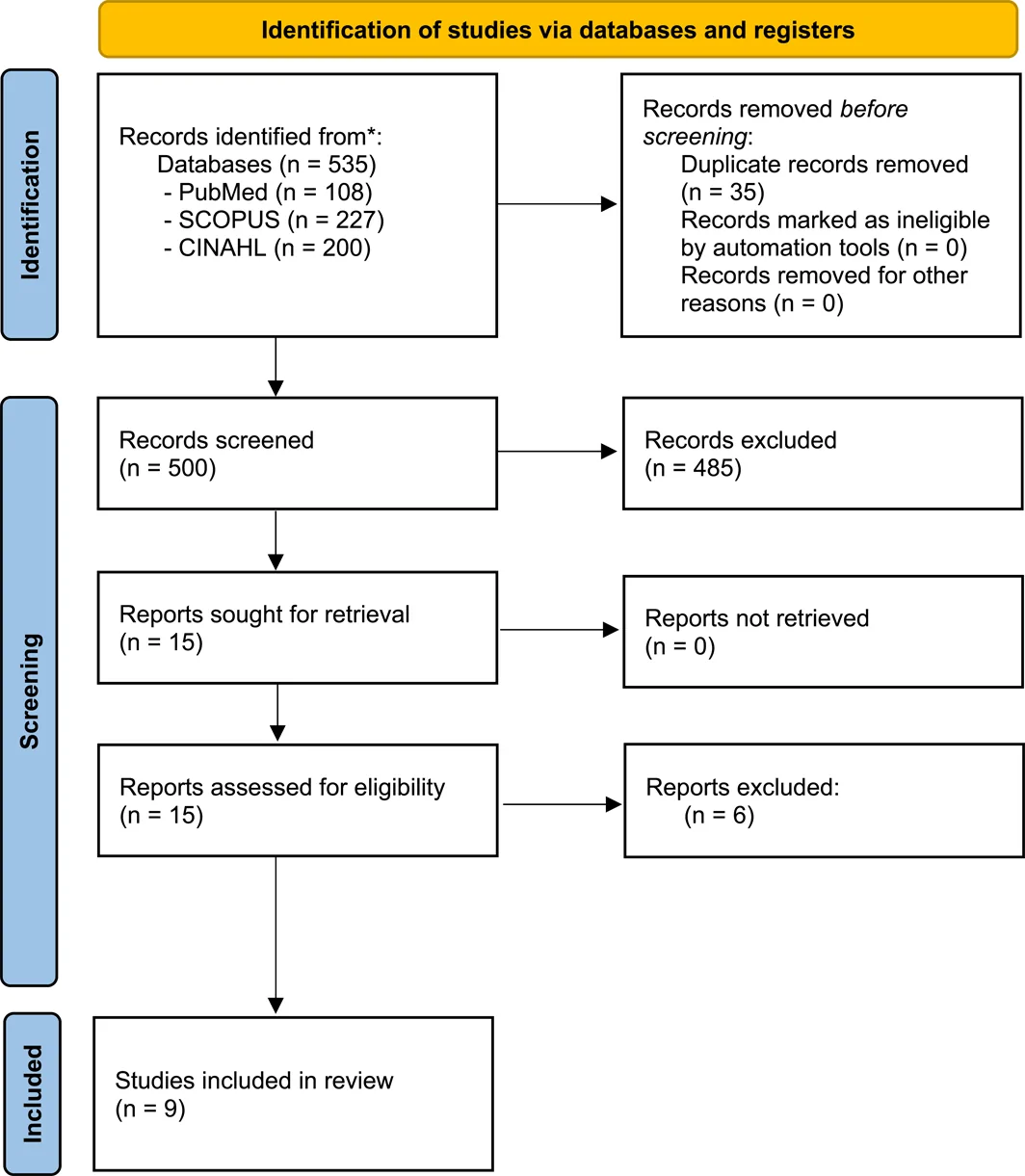
PRISMA flow diagram illustrating the process of screening and selecting literature. *Consider, if feasible to do so, reporting the number of records identified from each database or register searched (rather than the total number across all databases/registers). **If automation tools were used, indicate how many records were excluded by a human and how many were excluded by automation tools. *Source:* Page MJ, et al. BMJ 2021; 372:n71. doi: 10.1136/bmj.n71. This work is licensed under CC BY 4.0. To view a copy of this license, visit https://creativecommons.org/licenses/by/4.0/.

### 2.2 Inclusion and Exclusion Criteria

Studies were included if they were peer‐reviewed research articles published in English between 2020 and 2025, focusing on adult patients ( ≥ 18 years) undergoing elective foot and/or ankle surgery for non‐traumatic conditions. Eligible studies reported at least one quantifiable postoperative complication and included associated risk factors, patient outcomes, or mitigation strategies. Included study designs comprised prospective and retrospective cohort studies, case‐control studies, and case series involving 10 or more patients.

Exclusion criteria included systematic reviews, meta‐analyses, case reports or case series involving fewer than 10 patients, narrative reviews, editorials, opinion pieces, and conference abstracts. Studies were also excluded if they involved pediatric populations, focused on non‐surgical interventions, included trauma‐related surgical populations such as those undergoing fracture fixation, open reduction and internal fixation (ORIF), or other non‐elective procedures, lacked quantitative outcome data on postoperative complications, or employed surgical techniques not applicable to contemporary healthcare systems.

Although the initial database queries retrieved 535 articles, application of strict inclusion criteria (adult elective procedures, quantitative complication data, ≥ 10 patients per series, exclusion of trauma, pediatric, or outdated technique studies) yielded nine eligible papers representing a combined total of 20,455 patients. The 2020–2025 window was chosen to capture contemporary evidence that reflects current practice and outcome reporting standards.

### 2.3 Data Extraction and Quality Assessment

Authors D.M., L.S., and C.P. independently performed data extraction and quality assessment for the included studies. Extracted data included: first author, year of publication, study title, study design, population characteristics, surgical procedure(s), types and incidence of reported complications, analysis of risk factors, and any reported outcomes related to mobility, mental health, or quality of life. Each study was assigned a level of evidence based on *The Journal of Bone & Joint Surgery's* evidence hierarchy [38], which classifies studies as follows:

- Level I: High‐quality randomized controlled trials (RCTs) or systematic reviews/meta‐analyses of RCTs.
- Level II: Lesser‐quality RCTs or prospective cohort studies with comparison groups.
- Level III: Retrospective cohort studies or case‐control studies.
- Level IV: Case series or retrospective studies without comparison groups.
- Level V: Expert opinion, narrative reviews, or basic science without clinical correlation.

In addition to evidence grading, the risk of bias was assessed using the National Heart, Lung, and Blood Institute (NHLBI) Study Quality Assessment Tools [39]. The tool selected was matched to the study design (e.g., cohort, case‐control, or case series). Each study was rated across a standardized set of criteria, including study population, comparability of groups, exposure and outcome assessment, statistical adjustment for confounding factors, and loss to follow‐up. Based on the overall number and significance of methodological strengths and weaknesses, studies were categorized as follows:

- Good (low risk of bias): Most or all criteria met; minimal threats to internal validity.
- Fair (moderate risk of bias): Some criteria not met or unclear; potential for bias that could affect results.
- Poor (high Risk of bias): Many criteria unmet or unclear; significant concerns regarding validity or reliability of findings.

Disagreements in data extraction or quality ratings were resolved through discussion among the reviewers.

### 2.4 Heterogeneity and Rationale for Narrative Synthesis

Substantial heterogeneity across the nine included studies precluded quantitative synthesis. Patient populations ranged from professional dancers undergoing posterior ankle surgery [40] to patients with complex ankle/hindfoot reconstructions or total ankle arthroplasty with deformity correction [41, 42]. Procedural scope and techniques varied widely, including arthroscopic versus open ligament reconstruction [43] and different osteotomy alignment strategies [42]. Outcome definitions were inconsistent—for example, “any infection” versus “deep infection” [41], aggregated wound complications versus specific healing or dehiscence events [44], and reoperation reported as either “any secondary surgery” or procedure‐specific events [42]. Timeframes for outcome capture also differed from early postoperative outcomes to later complications such as nonunion and revision arthrodesis [42]. Perioperative protocols were likewise inconsistent, with variations in rehabilitation pathways, weight‐bearing progression, imaging review practices, and analgesia regimens. Given these clinical and methodological differences—spanning populations, procedures, outcome definitions, and follow‐up windows—pooling effect estimates was deemed inappropriate due to the risk of generating misleading summary statistics. A narrative synthesis was undertaken to preserve the nuance of individual study findings.

## 3 Results

### 3.1 Study Selection and Characteristics

Nine studies met the inclusion criteria (Table 1). Most were retrospective cohort studies (7/9), with two retrospective case series. Levels of evidence clustered at Level III (7/9) and Level IV (2/9), with no Level II studies; all were rated “Fair” on quality assessment. Studies originated primarily from the United States (*n* = 5) and Germany (*n* = 3), with one additional report from Taiwan. Sample sizes ranged from 20 [43] to 17,996 [44], for a combined total of 20,455 participants across all studies. Procedures encompassed hallux valgus correction (i.e., Lapidus), ankle arthrodesis and total ankle arthroplasty, ligament reconstruction, and dancer‐specific hindfoot procedures (e.g., os trigonum excision). Of the 15 full‐text reports assessed for eligibility, six were excluded. Reasons for exclusion were: ORIF/trauma population (*n* = 1, identified during peer review); predominantly trauma or non‐elective population (*n* = 2); no quantitative complication data (*n* = 1); case series with fewer than 10 patients (*n* = 1); and conference abstract without accessible full text (*n* = 1).

**TABLE 1:** Characteristics of included studies on elective foot and ankle surgery (2020–2025).

Ref.	Authors/year	Study type	Key methodological features	Level of evidence	Quality assessment	Country	Journal	Article title
[45]	Shah et al. (2024)	Retrospective cohort study	Large database; adjusted analysis; potential selection bias; incomplete follow‐up	III	Fair	United States	European journal of orthopedic surgery & traumatology	A predictive algorithm for perioperative complications and readmission after ankle arthrodesis.
[44]	Crook et al. (2023)	Retrospective cohort study	Propensity‐matched national dataset; robust sample; limited variable definitions	III	Fair	United States	Orthopedic journal of sports medicine	Operative versus nonoperative treatment of acute achilles tendon rupture: A propensity score–Matched analysis of a large national dataset.
[41]	Newton et al. (2023)	Retrospective cohort study	Defined endpoints; adjusted for opioid exposure; small subgroup sizes	III	Fair	United States	Foot & ankle specialist	Preoperative opioid Use predicts poorer outcomes of total ankle arthroplasty and hindfoot fusions.
[46]	Shrake et al. (2023)	Retrospective cohort study	Controlled exposure/outcome; incomplete confounder control	III	Fair	United States	Foot & ankle specialist	Preoperative opioid therapy correlated with increased rate of complications in foot and ankle surgery.
[43]	Yang et al. (2022)	Retrospective cohort study	Clearly described technique comparison; very small sample	III	Fair	Taiwan	The orthopedic journal of sports medicine	Chronic lateral ankle instability treated with tendon allografting: A preliminary comparison of arthroscopic and open Anatomic ligament reconstruction.
[47]	Ettinger et al. (2021)	Retrospective cohort study	Defined outcome measures; no blinding; moderate follow‐up	III	Fair	Germany	Foot & ankle international	Comparison of isolated screw to plate and screw fixation for tarsometatarsal arthrodesis including clinical outcome predictors.
[48]	Meyer et al. (2021)	Retrospective cohort study	Nutritional risk stratified; good data completeness; limited external validity	III	Fair	Germany	Acta orthopaedica	Impact of malnutrition and vitamin deficiency in geriatric patients undergoing orthopedic surgery.
[42]	van der Plaat et al. (2022)	Retrospective case series	Detailed procedure description; no comparator; long follow‐up	IV	Fair	Germany	The foot	Medial malleolar osteotomy for the correction of tibiotalar varus deformity during total ankle arthroplasty: Results in 95 ankles.
[40]	Walsh et al. (2020)	Retrospective case series	Clearly defined cohort; limited sample size; descriptive only	IV	Fair	United States	Orthopedic journal of sports Medicine	Surgical outcomes of Os trigonum syndrome in dancers: A case series.

*Note:* This table summarizes key features of the nine studies included in the review, ordered by level of evidence and reverse chronological year. Extracted variables include reference identifier, author(s) and year, country of origin, study type, journal, study population/procedure focus, level of evidence (journal of bone & joint surgery hierarchy) [38], and quality assessment rating (NHLBI tool) [39].

Methodological quality was appraised using the NHLBI Study Quality Assessment Tools, applied according to the study design. All included studies were rated Fair, indicating a moderate risk of bias. Principal quality domains and limitations are summarized in Table 1. Common weaknesses were retrospective design, limited adjustment for confounding factors, and incomplete follow‐up.

In addition to complication reporting, several studies included validated patient‐reported outcome measures (PROMs) such as FAOS, FAAM, or anxiety/pain scales [43, 45]. These outcomes are complementary but were not consistently reported across the literature.

### 3.2 Complication Types and Incidence

Commonly reported complications spanned wound‐related problems, infection, hardware issues, bone‐healing disturbance, nerve injury, pain/stiffness, systemic events, and late biomechanical sequelae (Tables 2, 3, 4). Only four of the nine studies reported measures of variability such as standard deviations or confidence intervals, limiting formal pooling of incidence data.

- Infection. Reported incidence ranged from 0.3% in a large matched ATR cohort (*n* = 17,996; operative 0.3% vs. nonoperative 0.2% [44]) to 12.5% in a mixed TAA/hindfoot‐fusion cohort (*n* = 190; “any infection” 12.5%; deep 7.5% [41]).
- Wound healing. Delayed healing/dehiscence ranged from 0.5% [44] to 10.0% [41].
- Hardware‐related. Implant failure/removal events ranged from 1.0% to 15.5% [47].
- Nonunion/bone healing failure. Rates ranged from 0.8% [46] to 18.7% [41].
- Nerve‐related. From 0.2% following arthroscopic ligament reconstruction [43] to 10.0% sural nerve injury in dancers [40].
- Pain/stiffness. From 0.8% postoperative stiffness [43] to 18.7% persistent pain [40].
- Systemic medical events. Where reported, combined medical complications reached 7.4% in malnourished geriatric patients versus 3.6% in nourished controls (*n* = 599) [48].
- Biomechanical sequelae. From 0.8% postoperative stiffness [43] to 6.8% scar‐tissue discomfort [40].

**TABLE 2:** Infection, wound healing, hardware, and bone‐healing complications.

Ref.	Authors/year	Level of evidence	Number of patients included	Type of complication (incidence)			
				Infection	Wound healing	Hardware‐related	Nonunion/Bone healing failure
[45]	Shah et al. (2024)	III	1084	Wound infection (NR)	Wound infection (NR)	—	—
[44]	Crook et al. (2023)	III	17,996	Operative (0.3%) versus nonoperative (0.2%), *p* = 0.2165	Operative (0.5%) versus nonoperative (0.2%), *p* = 0.0007	—	—
[41]	Newton et al. (2023)	III	190	Any infection: (12.5% vs. 3.3%; *p* = 0.036) superficial: (5.0% vs. 2.0%; *p* = 0.284) deep: (7.5% vs. 1.3%; *p* = 0.064)	Wound breakdown (dehiscence/separation): (10.0% vs. 4.7%; *p* = 0.248).	Hardware removal: (10.0% vs. 7.3%; *p* = 0.524)	Yes. (16.1% vs. 18.7% *p* = 0.750)
[46]	Shrake et al. (2023)	III	326	Yes (0.6% in opioid‐exposed vs. 3.8% in naïve patients; *p* = 0.055)	Delayed wound healing: (4.5% in exposed vs. 3.8% in naïve patients; *p* = 0.731)	Hardware removal: *6.1% in exposed versus 3.8% in naïve patients; *p* = 0.494)	Yes (6.1% in exposed vs. 0.8% in naïve patients; *p* = 0.017)
[43]	Yang et al. (2022)	III	20	Superficial infection: (10%) arthroscopic; (10%) open. Deep infection: (0%) both groups. Timing: 1 month	—	—	—
[47]	Ettinger et al. (2021)	III	101	Yes (NR)	Yes (9.7%)	Implant failure: TMT I (8.9%), TMT II (1.0%), TMT III (4.3%) Implant removal (15.5%)	Yes (12.6%)
[48]	Meyer et al. (2021)	III	599	—	Yes (7.4% vs. 1.3%; malnourished vs. normal; *p* = 0.001)	Yes (4% vs. 6%; malnourished vs. normal; *p* = 0.5)	—
[42]	Van der Plaat et al. (2022)	IV	95	Deep infections (4.2%)	Superficial wound debridement (2.1%)	Medial malleolar hardware removal (5.3%)	Medial malleolar nonunion (1.1%)
[40]	Walsh et al. (2020)	IV	44	—	—	—	—

*Note:* Reported incidence of surgical site infection, wound‐healing problems, hardware‐related complications, and nonunion/bone‐healing failure across included studies. Data are shown as percentages where available, with timing noted if explicitly reported.

**TABLE 3:** Reoperation, readmission, pain/stiffness, and nerve‐related complications.

Ref.	Authors/year	Level of evidence		Number of patients included	Type of complication (incidence)			
					Reoperation or revision	Readmission	Pain and stiffness	Nerve‐related
[45]	Shah et al. (2024)		III	1084	—	Yes (NR) Timing: 30 days	Pain (NR) Timing: 30 days	
[44]	Crook et al. (2023)		III	17,996	1‐year (1.1% for operative vs. 1.3% for nonoperative; *p* = 0.1201); 2‐year (1.9% for operative vs. 2% for nonoperative; *p* = 0.2810)	—	Stiffness: Operative (1.0%) versus nonoperative (1.2%), *p* = 0.3517	Yes (NR)
[41]	Newton et al. (2023)		III	190	Yes (22.5% vs. 10.7% *p* = 0.049)	—	—	—
[46]	Shrake et al. (2023)		III	326	Reoperation: (18.2% in exposed vs. 9.2% in naïve patients; *p* = 0.038)	—	—	—
[43]	Yang et al. (2022)		III	20	—	—	Stiffness of the ankle: Arthroscopic (0%); open (10%)	Paresis/numbness (sural or superficial peroneal): Arthroscopic (0%); open (10%).
[47]	Ettinger et al. (2021)		III	101	Revision surgery (25.2%)	—	Metatarsalgia (10.7%)	—
[48]	Meyer et al. (2021)		III	599	Reoperation (13% vs. 5.5%; malnourished vs. normal; *p* = 0.01) Timing: within 90 days	Yes (8.8% vs. 5.5%; malnourished vs. normal; *p* = 0.3) Timing: within 90 days	—	—
[42]	Van der Plaat et al. (2022)		IV	95	Any secondary surgery: (44%); total secondary procedures (55.8%) Any revisions (29%); aseptic revisions (25.3%); septic revisions (4.2%)	—	—	—
[40]	Walsh et al. (2020)	IV		44	—	—	—	Sural nerve injury (4.5%)

*Note:* Reported rates of reoperation or revision procedures, hospital readmissions, pain or stiffness outcomes, and nerve‐related complications across included studies. The timing of reoperation or readmission is noted when specified.

**TABLE 4:** Systemic, biomechanical, psychological, and other complications.

Ref.	Authors/year	Level of evidence	Number of patients included	Type of complication (incidence)			
				Systemic medical	Joint or biomechanical sequelae	Psychological factors	Other/Unspecified
[45]	Shah et al. (2024)	III	1084	—	Yes (NR)	—	—
[44]	Crook et al. (2023)	III	17,996	VTE: Operative (1.3%) versus nonoperative (1.5%); *p* = 0.3403	Heterotopic ossification: Operative (7.1%) versus nonoperative (6.3%); *p* = 0.0088	—	—
[41]	Newton et al. (2023)	III	190	Ulceration (2.5% vs. 0.7%; *p* = 0.378).	—	—	—
[46]	Shrake et al. (2023)	III	326	Ulceration (4.5% in exposed vs. 0.4% in naïve patients; *p* = 0.027)	—	—	—
[43]	Yang et al. (2022)	III	20	—	—	—	—
[47]	Ettinger et al. (2021)	III	101	—	Secondary degenerative changes (3.9%); arthritis sesamoids (3.9%)	—	—
[48]	Meyer et al. (2021)	III	599	MI, heart failure, arrhythmias, pneumonia, renal failure combined (7.4% vs. 3.6%; malnourished vs. normal; *p* = 0.1)	—	—	Falls (8.4% vs. 2.9%; vitamin D severe deficiency vs. ≥ 12 ng/mL; *p* = 0.006) DVT (1% vs. 0.4%; vitamin D severe deficiency vs. ≥ 12 ng/mL) PE: (1% vs. 0.2%; vitamin D severe deficiency vs. ≥ 12 ng/mL) Clavien–Dindo IV° (7.4% vs. 2.4%; vitamin D severe deficiency vs. ≥ 12 ng/mL; *p* = 0.03)
[41]	Van der Plaat et al. (2022)	IV	95	—	Impingement debridement: non‐revision (4.2%); preceding later arthrodesis (1.1%)	—	Other non‐revision procedures included calcaneal osteotomy, lateral ligament reconstruction, MTP resection arthroplasty (NR)
[40]	Walsh et al. (2020)	IV	44	—	Mild peroneus brevis tendinitis (2.3%)	—	Scar‐tissue discomfort (6.8%)

*Note:* Reported systemic medical complications (e.g., cardiovascular, respiratory, renal), joint or biomechanical sequelae, psychological factors, and other/unspecified events. Examples include heterotopic ossification, scar‐tissue discomfort, or anxiety. Values represent percentages where provided.

To provide a simplified overview, all complications were also consolidated into a single summary table categorized by timing (peri‐operative, early, and late); Table 5.

**TABLE 5:** Consolidated overview of peri‐operative, early, and late complications reported across included studies.

Timing category	Common complications	Reported range (%)	Representative studies
Peri‐operative (intra‐/immediate post‐op)	Anesthetic events, bleeding, intra‐operative fracture	0.1–1.0	[41, 43]
Early (≤ 90 days)	Infection (0.3–12.5), wound breakdown (0.5–10.0), DVT/PE (0.2–1.5), early pain/stiffness	0.2–12.5	[41, 44, 48]
Late (> 90 days)	Non‐union (0.8–18.7), hardware removal (3.8–15.5), nerve injury (0.2–10.0), chronic pain/stiffness (0.8–18.7), degenerative/biomechanical sequelae (≤ 7)	0.2–18.7	[40, 41, 42, 47]

### 3.3 Timing of Complications

Timing was explicitly reported in 4 of 9 studies. Readmissions, where reported, were tracked within 90 days [48].

### 3.4 Reoperation and Readmission

Reoperation/revision was reported heterogeneously but ranged from 9.2% in opioid‐naïve patients [46] to 44% after TAA with deformity correction [42], the latter detailing total revisions 29%, aseptic 25.3%, and septic 4.2%. Readmission was inconsistently reported; a geriatric cohort noted 8.8% versus 5.5% (malnourished vs. nourished; within 90 days) [48].

### 3.5 Risk Factors for Complications

Risk factors were multifactorial and included patient‐related, surgical, and postoperative domains (Table 6).

- *Patient‐related*. Higher BMI/obesity was recorded as a risk feature in multiple cohorts [44, 46, 47].
- *Surgical/technique.* Reported contributors included surgical approach (open versus endoscopic) [40]; case mix/complexity (teaching‐hospital proxy) [45]; fixation technique [47], and history of prior ankle surgery [42].
- *Peri‐/postoperative factors.* Preoperative opioid exposure [41, 46] and postoperative opioid use [41] were captured in several datasets, and postoperative soft‐tissue issues (e.g., wound problems/heterotopic ossification) were also noted [44].

**TABLE 6:** Risk factors for postoperative complications in elective foot and ankle surgery.

Ref.	Authors/year	Level of evidence	Number of patients included	Type of risk factor			
				Demographic	Comorbidity	Surgical	Postoperative
[45]	Shah et al. 2024	III	1084	Teaching hospital status (as proxy for case mix and complexity); age	Diabetes, PVD, renal failure, cardio‐respiratory failure, obesity, musculoskeletal infection, history of fracture (hip/long bone)	Teaching hospital (as proxy for case mix and complexity)	—
[44]	Crook et al. 2023	III	17,996	Smoking	Diabetes; obesity	Operative treatment	Wound; ossification
[41]	Newton et al. 2023	III	190	Age; mental illness	Renal disease, ASA score	Preoperative opioid use	Postoperative opioid use
[46]	Shrake et al. 2023	III	326	Higher BMI	Renal disease; cardiovascular disease; ASA score	Preoperative opioid exposure	—
[43]	Yang et al. 2022	III	20	—	—	Open surgery (vs. Arthroscopic)	—
[47]	Ettinger et al. 2021	III	101	Age; lower preoperative EFAS scores; higher BMI	Prior adjacent joint fusion	Fixation technique	—
[48]	Meyer et al. 2021	III	599	—	Malnutrition; vitamin D deficiency	—	—
[42]	van der Plaat et al. 2022	IV	95	—	—	History of prior ankle surgery	—
[40]	Walsh et al. 2020	IV	44	—	—	Approach (open vs. endoscopic)	Compensatory tendinitis

*Note:* Risk factors were grouped into four domains: demographic, comorbidity, surgical, and postoperative. Examples include age, BMI, smoking, diabetes, vascular disease, operative approach, fixation technique, and opioid exposure. Data are extracted directly from study reports; effect sizes were inconsistently provided and are therefore not presented here.

### 3.6 Strategies to Reduce Complications

Across studies, clinical and postoperative strategies were most commonly described (clinical: 9 studies; postoperative: 7), with fewer institutional/system‐level approaches (4) and limited prehabilitation reporting (1) (Table 7).

- *Clinical/intraoperative.* Examples included deformity correction and osteotomy alignment planning in TAA [42] and approach selection tailored to FHL pathology in dancers [40].
- *Postoperative protocols.* Structured rehabilitation was detailed for dancers [40]. A TAA/hindfoot cohort reported a standard opioid prescription [41].
- *Institutional/system‐level.* One large database study modeled teaching‐hospital status as a proxy for case‐complexity in risk stratification [45].
- *Screening/optimization.* Nutritional screening is recommended in geriatric patients undergoing orthopedic surgery [48]. Prehabilitation‐specific interventions were rarely detailed.

**TABLE 7:** Strategies to reduce postoperative complications in elective foot and ankle surgery.

Ref.	Authors/year	Level of evidence	Number of patients included	Type of strategy			
				Clinical	Prehabilitation	Postoperative	Institutional/System‐Level
[45]	Shah et al. (2024)	III	1084	Predictive risk stratification via XGBoost model	—	—	Teaching hospital status modeled as risk proxy
[44]	Crook et al. (2023)	III	17,996	Propensity score matching controlled for patient comorbidities	—	—	—
[41]	Newton et al. (2023)	III	190	—	—	Standard opioid prescription: 40 pills 5 mg oxycodone/acetaminophen	—
[46]	Shrake et al. (2023)	III	326	Identifying high‐risk patients, multimodal pain regimens	Counseling to reduce preoperative opioid use	Recommendation for regional anesthesia, pain optimization	—
[43]	Yang et al. (2022)	III	20	Arthroscopic debridement, antibiotic‐soaked graft, precise fluoroscopic guide‐pin placement	—	Day 1: Partial weight‐bearing in boot Week 2: Begin ROM Week 4: Full weight‐bearing in high‐ankle shoe Month 4: return to sports	—
[47]	Ettinger et al. (2021)	III	101	Plate + screw fixation significantly reduced nonunion rate	—	Boot NWB x2 weeks → PWB x4 weeks → full WB at 10–12 weeks	Radiographic and pedobarographic follow‐up
[48]	Meyer et al. (2021)	III	599	Nutritional screening recommended	—	—	—
[42]	van der Plaat et al. (2022)	IV	95	Medial malleolar osteotomy, correction of hindfoot alignment	—	Below‐knee cast, weightbearing at 2 weeks, follow‐ups at 6 weeks, 3 months, 1 year, and biannually	—
[40]	Walsh et al. (2020)	IV	44	Choice of posteromedial approach to better address FHL pathology	—	12‐week structured rehab program detailed in phases	—

*Note:* Strategies were grouped into four domains: clinical/intraoperative, prehabilitation, postoperative, and institutional/system‐level. Examples include predictive risk stratification, propensity score matching, structured opioid prescribing protocols, regional anesthesia counseling, and teaching‐hospital status used as a system‐level risk proxy. Data are extracted directly from study reports; the effectiveness of individual strategies was inconsistently quantified and is therefore not presented here.

### 3.7 Summary of Clinically Relevant Findings

1. Across 9 studies (total *n* = 20,455), complication profiles varied using procedure and patient mix. Infection ranged from 0.3% [44] to 12.5% [41]; wound problems from 0.5% [44] to 10.0% [41]; hardware removal from 3.8% [46] to 15.5% [47]; nonunion from 0.8% [46] to 18.7% [41]; and nerve‐related complications from 0.2% [43] to 10.0% [40]. Reoperation ranged from 9.2% [46] to 44% [42]. The timing of complications was inconsistently reported (3/9 studies) and readmissions or reoperations documented within 90 days [48]. Consistent risk patterns (higher BMI, diabetes, smoking, age; surgical approach/complexity; opioid exposure) suggest opportunities for targeted preoperative optimization, careful technique/approach selection, and structured postoperative pathways, although robust comparative testing remains limited in this evidence base.

## 4 Discussion

### 4.1 Principal Findings

This systematic review synthesized evidence from 9 studies (*n* = 20,455) examining complications following elective foot and ankle surgery, providing a descriptive synthesis of reported complications, risk factors, and mitigation strategies across a range of elective procedures. Commonly reported adverse events included wound problems, infection, nonunion, and hardware issues, with nerve injury, pain/stiffness, systemic events, and biomechanical sequelae also described. Implant removal clustered after Lapidus procedures, nonunion was most common following complex hindfoot reconstructions, and sural nerve injury was concentrated in dancers undergoing os trigonum excision. Frequently cited risk factors included diabetes, obesity, smoking, peripheral vascular disease, advanced age, poor glycemic control, revision surgery, and multisegmented reconstruction. Risk reduction strategies emphasized comorbidity optimization, tailored surgical approaches, precise operative technique, and structured rehabilitation and follow‐up. Notably, complex regional pain syndrome (CRPS), deep vein thrombosis (DVT), and pulmonary embolism (PE) were absent as reported measures despite their recognized clinical relevance, suggesting potential under‐recognition or under‐reporting in this evidence base.

### 4.2 Comparison With Previous Literature

The pattern of infection, nonunion, and hardware‐related problems aligns with broader orthopedic and podiatric experience and with procedure‐specific reviews (e.g., total ankle arthroplasty; hallux valgus) [49, 50]. Notably, within the articles reviewed, these events were not confined to high‐complexity reconstructions. For example, substantial hardware removal was reported in forefoot/midfoot surgery [47], while higher infection and nonunion were reported in a complex ankle/hindfoot cohort [41]. This is consistent with the view that anatomical and vascular constraints of the foot and ankle, combined with individual patient risk profiles, may outweigh nominal procedural “complexity” as the sole predictor of adverse outcome [50, 51].

Previous reviews have been limited to single procedures such as hallux valgus correction or total ankle arthroplasty. By contrast, the present analysis provides a descriptive synthesis of reported complications, risk factors, and mitigation strategies across a range of elective foot and ankle procedures, offering a cross‐procedural reference that may support evidence‐informed patient counseling, perioperative risk discussion, and benchmarking of surgical outcomes, albeit within the limitations of the heterogeneous evidence base.

### 4.3 Clinical Implications

#### 4.3.1 Preoperative Risk Assessment and Optimization

Diabetes, smoking, and higher BMI were repeatedly listed as risk factors [44, 45]. Although adjusted effect sizes were not consistently provided, the direction of risk is biologically plausible and consistent with external evidence: diabetes is associated with higher rates of surgical site infection after foot and ankle surgery [52], obesity increases wound complications [22], and smoking impairs fracture healing and fixation stability [53]. Nutritional status is also relevant [48]; higher medical complications and readmissions in malnourished geriatric patients have been reported, underscoring the value of targeted screening and optimization before elective procedures.

#### 4.3.2 Surgical Planning and Technique

Several studies emphasized technique choice and implant strategy: Yang et al. (2022) contrasted arthroscopic versus open ligament reconstruction (with differing stiffness profiles) [43]; Ettinger et al. (2021) described transfer metatarsalgia and implant removal after Lapidus procedures [47]; van der Plaat et al. (2022) reported reoperation burden in total ankle arthroplasty with deformity correction [42]. Collectively, these findings reinforce the importance of meticulous reduction, careful implant selection, and soft‐tissue respect across the elective spectrum. External evidence indicates that minimally invasive techniques may reduce wound complications: systematic reviews in hallux valgus surgery have reported fewer wound‐related complications with MIS compared with open approaches [4].

#### 4.3.3 Postoperative Pathways

Protocols were described for different populations: a structured 12‐week rehabilitation program for dancers [40], staged weight‐bearing and range‐of‐motion progressions after ligament reconstruction and arthrodesis procedures [42, 43, 47], and standardized opioid prescribing practices in TAA and hindfoot reconstructions [41]. While these illustrate plausible mitigation strategies, their comparative effectiveness was not uniformly assessed. External evidence, however, supports some approaches: randomized controlled and meta‐analytic studies in ankle fracture care demonstrate that early weight‐bearing can improve short‐term function without increasing complication rates [54, 55]. Taken together, these findings suggest that structured rehabilitation is a consistent and potentially modifiable component of postoperative care that may reduce complications and enhance recovery, though higher‐quality evidence is still needed.

### 4.4 Strengths and Limitations

A key strength of this review is its cross‐procedural scope, which provides a descriptive summary of reported complications, risk factors, and mitigation strategies across a range of elective procedures, an overview that narrower, procedure‐specific analyses do not offer. Limitations include heterogeneity in outcome definitions (e.g., differing thresholds and timing for “wound‐healing delay”), wide variation in follow‐up duration (ranging from weeks to years), and the predominance of retrospective single‐center designs. Where reported, inclusion of a median or mean follow‐up duration would have aided interpretation and comparability of complication rates across studies. Notably, there is paucity within the studies of directly reported PROMs, limiting insight into patient‐perceived recovery trajectories or satisfaction beyond clinical outcomes. These factors limit the feasibility of pooled estimation and causal inference, and may under‐represent minor complications that resolve without reoperation. Given the substantial variation in patient populations, procedures, outcome definitions, and follow‐up periods across the nine included studies, a narrative rather than quantitative synthesis was undertaken to avoid misleading summary estimates. A further limitation was inconsistent reporting of variability measures (e.g., confidence intervals, standard deviations), precluding meta‐analytic aggregation. Importantly, given that only nine heterogeneous studies were included across a broad range of procedures and patient populations, the findings are best interpreted as a descriptive synthesis rather than a robust characterization of complication incidence or recurring patterns across elective foot and ankle surgery as a whole.

### 4.5 Future Directions

Future research would benefit from standardized definitions for key complications, stratification by both procedure type and risk category, and prospective multicenter designs to improve generalizability. Trials assessing targeted perioperative optimization, particularly diabetic control, smoking cessation, and enhanced wound care protocols, are warranted to determine their effect size across different elective procedures. Integration of patient‐reported outcome measures (PROMs) will also provide a more complete understanding of how complications impact quality of life beyond binary clinical endpoints. In addition, future research should explore the impact of system‐level interventions such as surgical safety checklists, enhanced recovery after surgery (ERAS) protocols, and centralized outcome reporting frameworks—as these may contribute to standardizing care pathways and reducing complication rates across institutions. Procedure‐specific reviews applying the same systematic methodology employed here would also strengthen the evidence base by enabling more precise characterization of complication profiles within individual surgical categories.

## 5 Conclusions

Elective foot and ankle surgery is associated with a range of reported complications, most commonly wound problems, infection, nonunion, and hardware issues across the heterogeneous procedures and populations represented in this review. Common risk factors include diabetes, obesity, smoking, advanced age, and complex or revision surgery. Rehabilitation protocols have emerged as a recurring element of postoperative management, highlighting the potential role of structured weight‐bearing and range‐of‐motion progression in mitigating complications, though comparative evidence remains limited. Targeting modifiable risks, optimizing perioperative care, and adopting standardized definitions in future research may reduce complication rates and strengthen cross‐procedural evidence.

## Author Contributions

**Lia Spencer:** conceptualization, methodology, formal analysis, software, investigation, data curation, writing – original draft preparation, writing – review and editing, visualization. **Connor Phillips:** conceptualization, methodology, software, formal analysis, investigation, data curation, writing – original draft preparation, writing – review and editing, visualization. **Sifat Alam:** conceptualization, methodology, formal analysis, software, investigation, data curation, writing – original draft preparation, writing – review and editing, visualization. **Kit Ferguson:** conceptualization, methodology, formal analysis, software, investigation, data curation, writing – original draft preparation, writing – review and editing, visualization. **Richard Feinn:** conceptualization, methodology, formal analysis, writing – original draft preparation, writing – review and editing, visualization, resources. **Douglas McHugh:** conceptualization, methodology, formal analysis, software, investigation, data curation, writing–original draft preparation, writing – review and editing, visualization, resources, supervision, project administration. **Ewan Kannegieter:** conceptualization, methodology, formal analysis, software, investigation, data curation, writing–original draft preparation, writing – review and editing, visualization, resources, supervision, project administration. All authors have read and agreed to the published version of the manuscript.

## Funding

The authors have nothing to report.

## Ethics Statement

This study was reviewed by the Institutional Review Board of Quinnipiac University and deemed exempt from review, as it used publicly available, non‐identifiable data and did not meet the definition of human subjects research (45 CFR 46.110).

## Consent

The authors have nothing to report.

## Conflicts of Interest

The authors declare no conflicts of interest.

## Data Availability

The data supporting the findings of this study were derived from publicly available sources. All included studies are cited within the manuscript. Extracted data are available from the corresponding author upon reasonable request.

## Appendix 1 Screening Prompt

### ChatGPT Prompt for Systematic Review Abstract Screening

#### Objective Statement

To systematically review and synthesize global literature on complications following foot and ankle surgery, analyzing their incidence, associated risk factors, and impact on patient outcomes, including mobility, mental health, and quality of life. This review differentiated between elective and non‐elective procedures, compared outcomes across healthcare settings, and identified strategies for reducing complications.

#### Instructions provided to ChatGPT‐4o (OpenAI, May 2024 version):

The model was asked to categorize abstracts as INCLUDE, MAYBE, or EXCLUDE based on explicit inclusion and exclusion criteria.

#### Inclusion criteria:

- Population: Adults (≥ 18 years) undergoing elective foot and/or ankle surgery (e.g., bunionectomy, joint arthroplasty, joint fusion, tendon repair, minimally invasive/open procedures).
- Complications: At least one postoperative complication was reported and quantified (e.g., infection, hardware failure, nerve injury, vascular compromise, wound dehiscence, thromboembolism, chronic pain, etc.).
- Design: Prospective or retrospective cohort studies, case‐control studies, or large‐scale observational studies with robust methodology.
- Outcomes: Includes risk factors, patient outcomes (mobility, mental health, quality of life), or mitigation strategies.
- Timeframe: Published 2020–2025.
- Language: English.

#### Exclusion criteria:

- Non‐surgical treatments (e.g., physical therapy).
- Non‐elective or trauma surgeries (e.g., Achilles tendon rupture, fracture fixation).
- Pediatric populations (< 18 years).
- No complications were reported.
- Systematic reviews, meta‐analyses, case reports, narrative reviews, editorials, or conference abstracts without full text.
- Small case series (*n* < 10).
- Animal or in vitro studies.
- Technique‐only papers without outcome data.
- Studies without quantitative data on complications.
- Outdated techniques or geographically niche practices with limited global applicability.

#### Classification system:

- INCLUDE–Clearly meets all inclusion criteria.
- MAYBE–Unclear; full‐text required for clarification.
- EXCLUDE–Meets ≥ 1 exclusion criterion.

#### Task:

For each abstract, the model was instructed:

Please analyze the following abstract and categorize it as INCLUDE, MAYBE, or EXCLUDE, followed by a brief justification. Double‐check your work before reporting.
